# Pediatric Residents’ Perceptions of Potential Professionalism Violations on Social Media: A US National Survey

**DOI:** 10.2196/mededu.5993

**Published:** 2017-01-31

**Authors:** Rachel Dawkins, William D King, Beatrice Boateng, Michele Nichols, Bonnie C Desselle

**Affiliations:** ^1^ Department of Pediatrics Johns Hopkins School of Medicine Baltimore, MD United States; ^2^ Pediatric and Adolescent Medicine Johns Hopkins All Children's Hospital St Petersburg, FL United States; ^3^ Department of Pediatrics University of Alabama at Birmingham School of Medicine Birmingham, AL United States; ^4^ Department of Pediatrics University of Arkansas for Medical Sciences Little Rock, AR United States; ^5^ Department of Pediatrics Louisiana State University Health Sciences Center New Orleans, LA United States

**Keywords:** social media, professionalism, resident education, pediatrics, graduate medical education

## Abstract

**Background:**

The ubiquitous use of social media by physicians poses professionalism challenges. Regulatory bodies have disseminated guidelines related to physicians’ use of social media.

**Objective:**

This study had 2 objectives: (1) to understand what pediatric residents view as appropriate social media postings, and (2) to recognize the degree to which these residents are exposed to postings that violate social media professionalism guidelines.

**Methods:**

We distributed an electronic survey to pediatric residents across the United States. The survey consisted of 5 postings from a hypothetical resident’s personal Facebook page. The vignettes highlighted common scenarios that challenge published social media professionalism guidelines. We asked 2 questions for each vignette regarding (1) the resident’s opinion of the posting’s appropriateness, and (2) their frequency of viewing similar posts. We also elicited demographic data (age, sex, postgraduate year level), frequency of Facebook use, awareness of their institutional policies, and prior social media training.

**Results:**

Of 1628 respondents, 1498 (92.01%) of the pediatric residents acknowledged having a Facebook account, of whom 888/1628 (54.55%) reported daily use and 346/1628 (21.25%) reported using Facebook a few times a week. Residents frequently viewed posts that violated professionalism standards, including use of derogatory remarks about patients (1756/3256, 53.93%) and, much less frequently, about attending physicians (114/1628, 7.00%). The majority of the residents properly identified these postings as inappropriate. Residents had frequently viewed a post similar to one showing physicians drinking alcoholic beverages while in professional attire or scrubs and were neutral on this post’s appropriateness. Residents also reported a lack of knowledge about institutional policies on social media (651/1628, or 40.00%, were unaware of a policy; 204/1628, or 12.53%, said that no policy existed). A total of 372/1628 respondents (22.85%) stated that they had never received any structured training on social media professionalism.

**Conclusions:**

Today’s residents, like others of their generation, use social media sites to converse with peers without considering the implications for the profession. The frequent use of social media by learners needs to change the emphasis educators and regulatory bodies place on social media guidelines and teaching professionalism in the digital age.

## Introduction

Physicians are using social networking sites with increasing frequency. Recent reviews of social media use by physicians indicate widespread use in medical education [1] and for personal and professional purposes [2-4]. A review of the characteristics of physicians using social media indicated a high use by those under 35 years old practicing internal medicine, pediatrics, obstetrics and gynecology, and family medicine [5].

Social media technology offers great educational benefits with its ability to reach a vast audience instantaneously. Patients and families are using social media to connect with health care providers and to seek medical advice.

At the same time, these advanced tools bring challenges to our profession in the form of ethical dilemmas regarding proper physician-patient relationships, privacy concerns, and the portrayal of physicians on the Internet.

Several reports [6] have highlighted these concerns by documenting breaches of professionalism by practicing physicians, prompting regulatory and professional organizations, such as the US Federation of State Medical Boards (FSMB), to develop and disseminate guidelines related to the use of social media by physicians [7-10].

Resident trainees are particularly at increased risk of the consequences of using social media. Some experts have reported concerns that the current generation of residents, who have been coined the “digital native generation” (born after 1980), will apply guidelines about online professionalism differently from the older digital “immigrant” generation [11].

In fact, a recent study reported that pediatric program directors find lapses in online professionalism by pediatric residents to be quite common, with over half of the program and associate program directors reporting inappropriate postings by residents in the past year [12]. Similar to the FSMB, medical schools have realized the need for social media educational guidance to trainees, noting online behaviors such as violations of patient privacy, use of profanity, depiction of intoxication, sexual suggestiveness, and communication about the medical profession or patients in a negative tone [13].

To date, most of the studies related to physicians’ use of social media have largely focused on its use, and guidelines for helping physicians navigate the blurred lines. Previous research has elicited the opinions and concerns of US medical school deans, state medical boards, and pediatric clerkship directors and residency program directors regarding social media use by learners [12-15]. One recent study compared perceptions of pediatric residents with those of program directors using descriptors of online activity [16]. However, to our knowledge, none have directly surveyed trainees by using actual Facebook posts.

By conducting a national survey in the United States of all pediatric residents we sought to determine (1) residents’ perspectives on appropriate social media postings, and (2) the degree to which residents are exposed to postings that violate regulatory and professional organization guidelines for social media use.

## Methods

### Recruitment

In March 2013, we distributed an electronic survey via SurveyMonkey (SurveyMonkey, San Mateo, CA, USA) to members of the American Academy of Pediatrics Section on Medical Students, Residents and Fellowship Trainees (AAP SOMSRFT) (now the Section on Pediatric Trainees). At the time of this study, approximately 98% of all pediatric residents were members of AAP SOMSRFT. For the purposes of this study, we used responses from the pediatric and medicine-pediatric residents only (N=9850). The survey site was open for 3 weeks from March 5 to March 25. No reminder emails were sent. The survey was voluntary, and we offered an incentive to complete the survey in the form of a chance to win a cash prize.

### Survey Design

**Figure 1:**
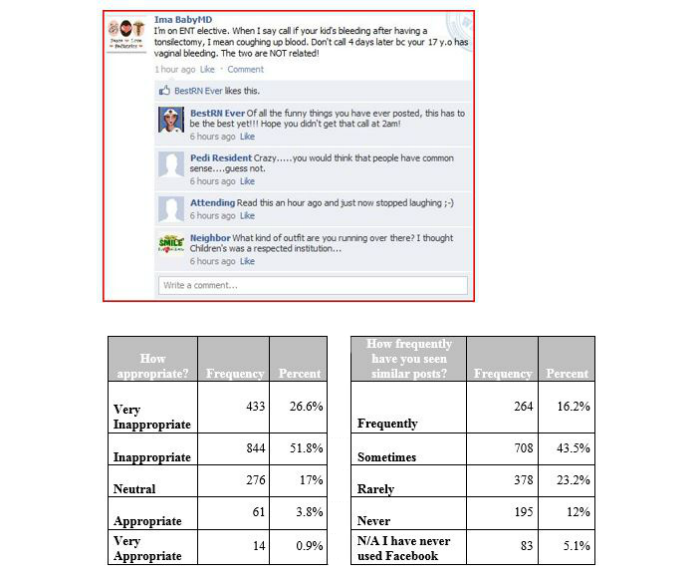
Vignette 1, depicting physicians' use of derogatory remarks about patients. N/A: not applicable.

**Figure 2:**
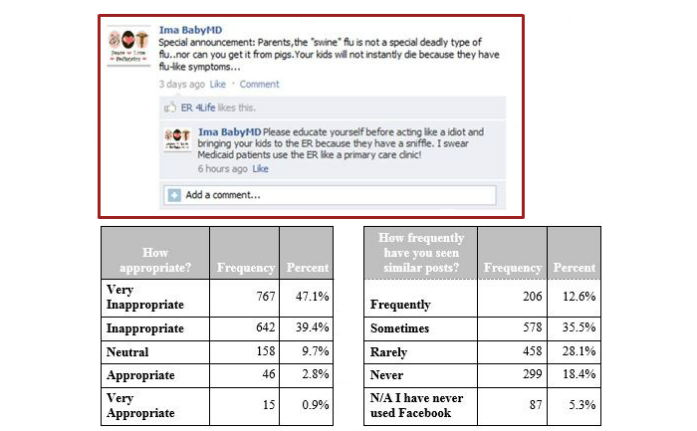
Vignette 2, depicting a physician's use of derogatory remarks about patients. ER: emergency room; N/A: not applicable.

**Figure 3:**
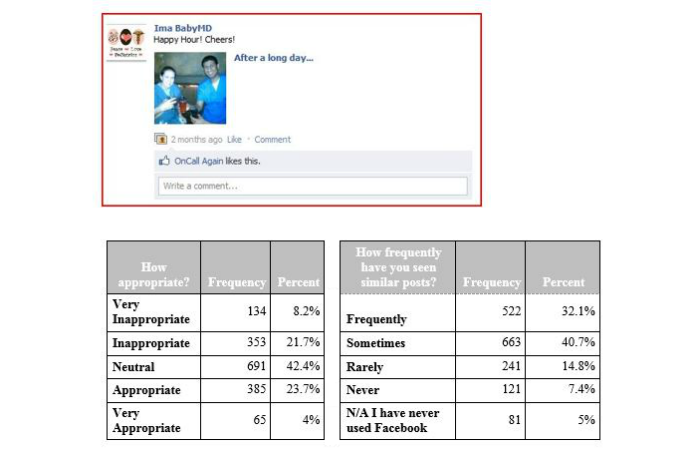
Vignette 3, depicting physicians wearing medical attire and consuming alcohol. N/A: not applicable.

**Figure 4:**
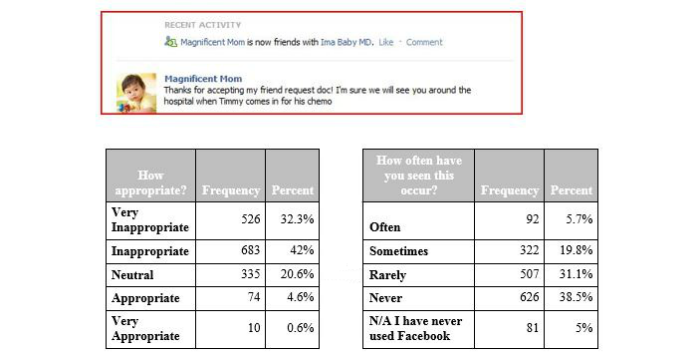
Vignette 4, addressing appropriate physician-patient boundaries. N/A: not applicable.

**Figure 5:**
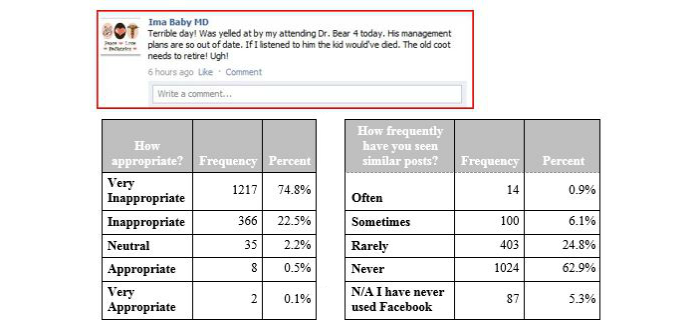
Vignette 5, depicting a physician's use of derogatory remarks about another physician. N/A: not applicable.

The survey consisted of 5 hypothetical postings from a resident’s personal Facebook page (Facebook, Inc, Menlo Park, CA, USA). We based these vignettes on our observations of actual postings by residents from their institutions and mirrored the main criteria used by state medical boards to discipline physicians for unprofessional behavior [7]. Among the vignettes, 3 depict physicians’ use of derogatory remarks about patients (vignettes 1 and 2) and about another physician (vignette 5); vignette 3 illustrates physicians wearing medical attire and consuming alcohol; vignette 4 addresses appropriate physician-patient boundaries (see Figure 1, Figure 2, Figure 3, Figure 4, and Figure 5). We tested the vignettes on a small focus group of early-career pediatric faculty at the primary author’s institution, Louisiana State University Health Science Center, which included both social media users and those without social media accounts. We refined the vignettes based on feedback from the focus group. The vignettes do not encompass all areas discussed in published social media guidelines but were chosen as those most commonly encountered by trainees.

Using a Likert format, we asked 2 questions for each vignette regarding (1) the resident’s opinion of the appropriateness of the posting, using a 5-point ordinal scale from “very inappropriate” to “very appropriate,” and (2) the frequency with which the resident had viewed similar posts, using a 4-point incremental scale from “frequently” or “often” to “never,” plus an additional “I have never used Facebook” option. We also elicited demographic data (age, sex, and postgraduate year), frequency of Facebook use, awareness of their institutional policies, and prior social media training.

### Analysis

We imported data from SurveyMonkey into Microsoft Excel 2007 (Microsoft Corporation, Redmond, WA, USA), in order to prepare the survey dataset for statistical and tabular analysis. The study dataset contained the responses of postgraduate years 1 through 4 training levels and chief residents. The data analysis was performed using Epi Info Version 7 (Centers for Disease Control and Prevention, Atlanta, GA, USA). We performed basic descriptive analyses of responses for each question and report the corresponding frequency for each question response.

The institutional review boards of the Louisiana State University Health Sciences Center, the University of Alabama School of Medicine, and the University of Arkansas for Medical Sciences approved this study as exempt from requiring participants’ consent. Funding for this study was provided through a grant from the Louisiana State University Health Sciences Center New Orleans Academy for the Advancement of Educational Scholarship.

## Results

### Demographics

We received responses from 1628 pediatric residents (of 9850 surveyed; 16.53% participation rate). Of these, 92.01% (1498/1628) acknowledged having a Facebook account, of whom 888 (54.55%) reported daily use and 346 (21.25%) reported using Facebook a few times a week (Table 1).

**Table 1:** Frequency of Facebook use among 1628 pediatric residents.

Frequency	n	%
Daily	888	54.55%
A few of times a week	346	21.25%
A few times a month	130	7.99%
Rarely	145	8.91%
Never	119	7.31%

The total sample of 1628 residents included 1205 women (74.02%) and 423 men (25.98%). Mean age of the respondents using Facebook was 30 years (median 29 years; interquartile range 27-33 years). Mean age of the respondents who did not have a Facebook account was 31 years (median 30 years; interquartile range 25-37 years). Respondents were distributed throughout all postgraduate levels, with 554 (34.03%) in postgraduate year 1; 456 (28.01%) in year 2; 407 (25.00%) in year 3; and 211 (12.96%) in year 4 or chief residents.

Of note, there was no statistically significant difference in responses to the vignettes between Facebook users and non-Facebook users, nor was there a statistically significant difference between responses of various postgraduate year levels. Therefore, we report all responses in aggregate below.

### Analysis

In vignettes 1 and 2 depicting physicians’ use of derogatory remarks about patients (Figure 1, Figure 2) and about attending physicians (Figure 5), the majority of the residents properly identified these posting as inappropriate. However, the residents reported often seeing something similar (972/1628, 59.71% for vignette 1 and 784/1628, 48.16% for vignette 2 responding often and sometimes), but not vignette 5 (1427/1628, 87.65% rarely and never).

The third vignette (Figure 3) shows physicians drinking alcoholic beverages while in professional attire (scrubs). On this very often viewed posting (1215/1628, 74.63%), most residents were neutral (691/1628, 42.44%), with an even distribution toward appropriate and inappropriate.

In the fourth vignette (Figure 4), the resident accepts a friend request from a mother of a patient. The majority of residents recognized this as inappropriate (1209/1628, 74.26%) and as rarely or never seen (1133/1628, 69.59%).

We asked residents about their knowledge of the presence of social media policies at their institutions, pediatrics departments, or residency programs. Almost half of respondents (765/1628, 46.99%) said that their institution did have a policy. However, almost as many (651/1628, 39.99%) were unsure whether their institution, department, or program had a social media policy in place. Residents were also asked about any formal training on appropriate use of social media, and 418 respondents stated that they had never received any structured training on social media professionalism.

The most common method for training was in-person discussions by program leaders (n=706 responses), followed by formal lectures and discussions by hospital administration (n=458) or risk management personnel (n=402 responses). Simulation was the training experienced by a small group of the respondents (n=53). Other methods of training mentioned in free-text answers included prior training in medical school but not during residency, Web-based modules, and emails from superiors of the program’s social media policy and about instances of inappropriate social media use. Another 2 comments indicated that training shouldn’t be needed, as online professionalism is the “common sense of being an adult.”

## Discussion

### Principal Findings

This study is, to our knowledge, the first to report a US national survey of pediatric residents’ perspectives using simulated physicians’ Facebook postings. Residents could identify some inappropriate content but reported being frequently exposed to unprofessional posts. Despite widely disseminated guidelines on the professional use of social media content, the data show that these professionalism standards are being violated as reported previously [12,16].

Residents did recognize the inappropriate scenarios as such in 4 of the 5 vignettes. The disconcerting exception is vignette 3, where 70% of residents were neutral about or comfortable with a post depicting physicians drinking alcohol while in medical attire. A recent study found that 40% of state medical boards would consider investigating a physician, with similar postings, for breaches of professional conduct [17]. While wearing scrubs when dining at a restaurant or bar is not necessarily a breach of professionalism, patients, colleagues, and the public may perceive the physician to be working while under the influence of alcohol. Residents, like many of the digital native generation, may not consider the future implications for career, professional standing, future job searches, etc, because Internet posts are “forever,” leaving a digital footprint behind [18].

Regulatory groups discourage entering into an electronic “friendship” with patients (vignette 4) [6], and our study respondents recognized it as inappropriate, but to a lesser degree (around 70%) than published data on program directors’ opinions (99% disapproval) [12]. Physicians should use the same guidelines in entering digital conversations as they would in real life and consider that shared personal information may cloud the typical boundary of the physician-patient relationship. Residents should continue to be educated on this issue, as patients may make these types of “friend” requests to an independent practitioner more frequently in an established, longer-term physician-patient relationship.

Residents’ being exposed to unprofessional social media posts, as we report, may increase their propensity to model this behavior. Making disparaging comments about patients and other health care providers has no place in the dialogue of our profession and will undermine the public’s respect. Physicians need to be cognizant that comments about patient experiences, as in vignettes 1 and 2, can be viewed as a breach of confidentiality, even if no personal identifiers are included, thus undermining the public’s trust.

Our data show that a remarkably high percentage (92%) of responding residents use Facebook, with over 50% using it daily and another 20% using it at least once a week. This mirrors data from the general population, where 59% of adult respondents to a Centers for Disease Control and Prevention survey [5] and 74% of respondents to a Pew survey [19] reported use of social networking sites, with the highest rate being among 18- to 29-year-olds, the age group encompassing most medical residents. The prevalence of use of interactive Web technology by these learners underscores the need for social media education by medical educators, professional organizations, and regulatory groups. Education should not be limited to adherence to guidelines but should include what actions residents should take when they observe guidelines being violated by others [16]. Providing an anonymous, safe process for reporting, investigating, and addressing unprofessional behaviors online could lead to corrective actions being taken before state medical boards would intervene. Most medical schools have policies, guidelines, and processes for addressing professionalism at work. Those processes could be modified to include unprofessional behavior online.

### Limitations and Benefits

There are several limitations to our study. Although a large number of residents responded to this survey, the results represent only 16.53% of all pediatric residents who are members of the AAP SOMSRFT. We attribute this to our inability to send reminder emails to nonresponders.

The study focused only on pediatric and medicine-pediatric residents. While the vignettes were not necessarily specific to pediatrics, the results may not be generalizable to all residents. The possibility that nonresponders were not Facebook users must be considered and could have skewed the results. In addition, physicians and health care professionals use other user-generated content sites, but we did not focus on these sites. Our questions were limited to 5 scenarios, which does not represent all potential violations that are enumerated by the FSMB social media guidelines. This self-reported study might also have been subject to recall bias.

This type of study has several benefits. As with case-based learning of medical diseases, the use of real posts would enhance the relevance to learners, stimulate greater discussion, and enhance the acceptability of teaching social media professionalism compared with simply providing a list of *do*’s and *don’t*’s per published guidelines. Also, these results pinpointed generational and controversial areas, which can guide curriculum design. Due to the rapidly changing nature of the use of technology in medicine, follow-up studies would be useful to see whether lessons are learned and opinions evolve over time. Future studies may also compare learners of various levels versus attending physicians.

### Conclusion

A high percentage of residents reported viewing and, in some instances, not recognizing unprofessional posts. This highlights the need for further education of residents about the potential hazards of online postings in order for the continued high standards of professional behaviors to be upheld by the next generation of physicians.

## Abbreviations

AAP SOMSRFT: American Academy of Pediatrics Section on Medical Students, Residents and Fellowship Trainees
FSMB: Federation of State Medical Boards
